# Macrophage dysfunction in Psoriatic Arthritis

**DOI:** 10.1186/1479-5876-9-S2-P59

**Published:** 2011-11-23

**Authors:** Mark H Wenink, Kim CM Santegoets, Lenny van Bon, John Butcher, Wim B van den Berg, Piet LCM van Riel, Iain B McInnes, Timothy RDJ Radstake

**Affiliations:** 1 Dept. of Rheumatology, Nijmegen Center of Infection, Inflammation and Immunity (N4i) and Nijmegen Centre for Molecular Life Science (NCMLS), Radboud University Nijmegen Medical Centre, Nijmegen, The Netherlands; 2 Division of Immunology, Infection and Inflammation, Glasgow Biomedical Research Centre, University of Glasgow, Glasgow, Scotland, UK

## Introduction

The pathogenesis of Psoriatic Arthritis (PsA) remains poorly understood. The underlying chronic inflammatory immune response is thought to be triggered by unknown environmental factors and might arise due to an impaired (innate) immune function by Dendritic Cells (DCs) [1]. Anti-inflammatory CD163^+^ type 2 macrophages (mf-2) are thought to have important functions in restoring immune homeostasis during an inflammatory response. Mf-2 are present in PsA synovium at high numbers. Why the immune response in PsA goes awry, despite the presence of these mf-2, is still largely undetermined.

## Aim

We aimed to determine whether mf-2 from PsA patients have an aberrant phenotype thereby contributing to the chronic inflammation occurring in PsA.

## Patients and methods

Mf-2 from 12 healthy controls and 12 PsA patients were analysed for their expression of various cell surface receptors as well as their cytokine production when exposed to a range of bacteria and single Toll-Like receptor (TLR) ligands. Differences between healthy control and PsA mf-2 involved in suppressing DC function were determined by stimulation assays and mixed leukocyte reactions.

## Results

CCR5 was significantly increased on mf-2 from PsA patients compared to healthy controls while the expression of CCR1, TLR2, TLR4 and CD14 was unaltered. Further research revealed that PsA mf-2 secreted more IL-6 upon incubation with heat-killed *Escherichia coli* and *E. coli* lipopolysaccharide and were less efficient in phagocytosing *E. coli*. The production of TNFα and IL-10 was equivalent between the groups. The production of cytokines was equal between PsA and healthy control mf-2 upon the stimulation with *M. tuberculosis*, *P. gingivalis* or TLR2 or TLR7/8 ligands. In addition, PsA mf-2 were less capable of suppressing cytokine production by DCs compared to mf-2 from healthy controls. Further reflecting their disordered function was the finding that in T cell assays PsA mf-2 are less capable of suppressing DC induced IL-17 and TNFα production by T cells.

## Conclusion

Anti-inflammatory CD163^+^ mf-2 from PsA patients have a clearly aberrant phenotype. This implicates mf-2 as an important defective cell type in PsA facilitating chronic inflammation instead of exerting their normal function of restoring immune homeostasis.

## References

[B1] Wenink MH Santegoets KC Butcher J van Bon L Lamers-Karnebeek FG van den Berg WB Impaired dendritic cell proinflammatory cytokine production in psoriatic arthritis Arthritis Rheum 2011 21811995 10.1002/art.30577

